# Psychosocial Burden of Women Who Are to Undergo Additional Diagnostic Procedures Due to Positive Screening for Cervical Cancer

**DOI:** 10.3390/cancers16203541

**Published:** 2024-10-20

**Authors:** Irena Ilic, Goran Babic, Aleksandra Dimitrijevic, Sandra Sipetic Grujicic, Vladimir Jakovljevic, Milena Ilic

**Affiliations:** 1 Faculty of Medicine, University of Belgrade, 11000 Belgrade, Serbia; 2 Department of Gynecology and Obstetrics, Faculty of Medical Sciences, University of Kragujevac, 34000 Kragujevac, Serbia; 3 Institute of Epidemiology, Faculty of Medicine, University of Belgrade, 11000 Belgrade, Serbia; 4 Department of Physiology, Faculty of Medical Sciences, University of Kragujevac, 34000 Kragujevac, Serbia; 5 Department of Epidemiology, Faculty of Medical Sciences, University of Kragujevac, 34000 Kragujevac, Serbia

**Keywords:** cervical cancer, screening, Papanicolaou smear, diagnostic procedures, psychosocial burden

## Abstract

**Simple Summary:**

Receipt of an abnormal Papanicolaou result often leads to a psychosocial burden. This study reported independent predictors of higher psychosocial burden prior to diagnostic procedures (colposcopy/biopsy/endocervical curettage) in women who received an abnormal Papanicolaou screening result. Worry in women with a positive Papanicolaou screening test before diagnostics is associated with oral contraceptives use, alcohol use, and low knowledge of the meaning of term precancerous, while satisfaction with information/support is linked to psychological distress. Providing uniform and explicit information about cervical cancer screening to better understand the meaning of the term dysplasia/precancerous and identifying women at risk of psychosocial burden may help protect against this potential harm in women who receive a positive cervical-cancer-screening result and may facilitate their intention to undergo further diagnostic procedures.

**Abstract:**

**Background/Objectives**: This study aimed to evaluate psychosocial burden and its associated factors in women who were referred for additional diagnostic procedures following receipt of a positive cervical-cancer-screening smear result. **Methods**: A cross-sectional study was performed in a consecutive cohort of only women who received an abnormal Papanicolaou screening result and therefore presented to a gynecologist for additional diagnostic examinations (colposcopy/biopsy/endocervical curettage) at the Clinic for Gynecology and Obstetrics of the Clinical Center. Multivariate linear regression was used for data analysis, with Bonferroni correction applied for multiple comparisons. **Results**: Significant independent predictors for the occurrence of psychosocial burden–worry in women with a positive Papanicolaou screening test before diagnostic procedures were the use of oral contraceptives (β = −0.174, *p* < 0.001), alcohol consumption (β = 0.188, *p* < 0.001), anxiety (β = −0.189, *p* = 0.001), high burden of depressive symptoms (β = 0.191, *p* = 0.001) and insufficient knowledge of the meaning of the term dysplasia/precancerous (β = −0.187, *p* < 0.001), according to the multivariate linear regression. The significant independent predictor for the occurrence of psychosocial burden–satisfaction with information/support in women with a positive Papanicolaou screening test before diagnostic procedures was psychological distress (β = −0.210, *p* = 0.001). **Conclusions**: Providing information in order to improve understanding of the term dysplasia/precancerous, as well as identifying which women are at risk of psychosocial burden, may help protect against this potential harm among women who receive a positive cervical-cancer-screening result and may facilitate their intention to undergo further diagnostic procedures.

## 1. Introduction

Worldwide, cervical cancer is the fourth most common cancer in women (with about 663,000 new cases; 6.9% of the total) in 2022 [1]. Among those, about 397,000 (60%) of all cervical cancer incident cases in the world are reported in Asian countries. In 2022, cervical cancer was the most frequently diagnosed cancer in women in 23 countries. Women in Africa showed the highest age-standardized rates in the incidence of cervical cancer. Differences in incidence can be explained by differences in the economic development of the country, differences in lifestyle, by religious and cultural differences, and socio-economic status [2,3,4]. However, the disproportionate burden of cervical cancer in developing countries is largely a result of the absence of mass screening programs [5,6].

Based on the World Health Organization estimates, cervical cancer is the fourth leading cause of cancer-related death in women (with about 349,000 deaths; 8.1% of the total) in 2022 [1]. Of those, about 200,000 (60%) of total cervical cancer deaths in the world are reported in Asian countries. Women in Africa had the highest age-standardized rates of cervical cancer mortality. In 2022, cervical cancer was the leading cause of cancer-related deaths in 39 countries.

The global survival rate for cervical cancer ranges between 50 and 70%, in particular in high-income America and Europe [7]. When detected at an early stage, cervical cancer is one of the most successfully treated malignancies [8]. The five-year survival rate for patients with cervical cancer diagnosed at a localized stage among women aged 15–44 years in the US is about 90% [9]. The overall survival rate varies from less than 30% in Africa to 70% in North America and northern Europe [9]. Nation-wide estimates of cervical cancer survival are still not available for Serbia.

In Serbia, cervical cancer screening is performed as an organized decentralized program [10]. The basic screening test is a cytological swab of the cervix (Papanicolaou test). From the moment of the implementation of organized screening in 2013 in Serbia, over 70,000 cervical cytology tests have been performed yearly, and about 0.9% are reported as abnormal.

For women, receiving a positive Papanicolaou smear result sometimes leads to increased stress [11], fear of cancer [12], concern about general health [13], fear of death [14], anxiety and depressive symptoms [15], fear for future offspring [16], and dissatisfaction with sexual intercourse and quality of life [17]. A recent study in Turkish women found that abnormal cytological findings together with HPV 16/18 positivity exacerbate sexual dysfunction [17]. Moreover, women often show a lack of knowledge about the Papanicolaou test, believing that the test is unnecessary or of no use, or believing that they are not at risk of developing cervical cancer [18].

Some authors estimate that 5–60% of women with a positive Papanicolaou test result are lost to follow-up [19]. Explanations for non-adherence to cervical-cancer-screening guidelines are numerous and include age, education, financial status, comorbidity, obesity, psychosocial issues, and marital status [12,20]. Although organized screening programs are effective in reducing cervical cancer incidence and mortality in a population, the psychosocial burden of cervical cancer screening can represent an important harm to the women involved.

Previous studies were mainly concerned with investigating the psychological effects associated either with the invitation to participate in cervical cancer screening, or with the consequences of the screening itself (including both women with an abnormal result of the screening test and those with a normal result), and also in patients with cervical cancer with the aim of monitoring the effects of therapy and survival [9,16]. Despite the importance of colposcopy and other additional diagnostic procedures for women with abnormal Papanicolaou screening results, little is known about the prevalence of psychosocial effects prior to diagnostic procedures, as well as predictors of these effects of diagnostic procedures. Several studies in the United Kingdom, the Netherlands, Finland, and Canada noted that the reasons for non-adherence to follow-up after an abnormal Papanicolaou test included increased levels of anxiety and depression, but the results are not consistent [11,12,13,16,18]. To date, most studies have been conducted in developed countries, while experiences from countries with limited resources are lacking or insufficient in the context of the burden that cervical cancer represents in these populations. In order to improve the course and outcome of the disease, further research into predictors of psychosocial stress before colposcopy and other diagnostic procedures is of great importance for women with a positive Papanicolaou test.

In this study, psychosocial burden is defined as a women’s subjective perception of the burden experienced and has to do with undergoing cervical cancer screening after the receipt of an abnormal Papanicolaou smear result and before undergoing additional diagnostic examinations. Psychosocial burden has both psychological (involving emotions such as concern, anxiety, uneasiness, and depression) and social properties. According to the stress theory of cognitive appraisal proposed by some authors [21], the physiological response of stress occurs if a situation is appraised as negative (in this study, diagnostic examination is evaluated as the main stressor). The specific tool, Process-Outcome-Specific Measure (POSM), was developed within the context of a trial of management of borderline and other low-grade abnormal smears (TOMBOLA) study to assess the psychosocial impact of a low-grade abnormal cervical smear result and the subsequent management [22]. POSM presents psychosocial burden as a construct encompassing two key domains that comprise the psychosocial burden felt by women wanting diagnostics, including worries (regarding cervical cancer, general health, the result of the next cytology test, and having sex) and satisfaction with information and support (relating to feeling well-enough informed, being satisfied with support from other people, and how the woman felt about herself) [20,22].

Data on the psychosocial burden in women regarding additional diagnostic procedures following abnormal Papanicolaou smear results are scarce. This study aimed to evaluate psychosocial burden and its associated factors among women who were referred for additional diagnostic procedures following receipt of a positive cervical-cancer-screening smear result.

## 2. Materials and Methods

### 2.1. Study Setting and Study Population

This study was carried out in Kragujevac, a city located 120 km west of the Serbian capital Belgrade, with a population of 200,000. The research was conducted in 2017. In Serbia, the National Cancer-Screening Guidelines recommend the use of the Papanicolaou test for the screening of cervical cancer, in women aged 20–65 years who are invited to undergo cervical cancer screening every three years. For those women who had a positive result, additional diagnostic procedures are recommended [10].

If the screening result returns positive, the doctor involved in the screening examinations informs the screening participant of the results and refers her to further diagnostic procedures that are to be attended in the next four to six weeks. Adequate communication means providing information about why the woman is referred to a special examination regimen or further diagnostics and allows the screening participant to ask questions at each stage. Also, the participant is informed that the definitive diagnosis is made by histopathological examination of the tissue sample obtained during the diagnostic procedures (consultative colposcopy/biopsy/endocervical curettage).

### 2.2. Study Design

This cross-sectional study was conducted in a cohort consisting of all consecutive women who received an abnormal Pap screening test result and therefore presented to a gynecologist for additional diagnostic examinations at the Clinic for Gynecology and Obstetrics of the Clinical Center.

### 2.3. Study Sample

The criteria for inclusion of subjects in the study were as follows: receiving a positive Pap test result and undergoing diagnostic procedures at the Gynecology and Obstetrics Clinic of the Clinical Center in Kragujevac, age from 20 to 65 years, fluency in speaking and reading Serbian, voluntary consent to participate in the study, and absence of exclusion criteria.

The criteria for excluding subjects from the study were as follows: previous cancer of the cervix or intervention on the cervix, age below 20 or over 65 years, pregnancy that occurred during the study, presence of psychiatric diseases, existence of diseases of the reproductive organs for which treatment was ongoing during the study, refusal to participate in the research, or presence of any other objective reason that prevents or hinders participation in the study.

### 2.4. Sample Size Calculation

The sample size was determined based on the data of a multicenter study that studied the psychosocial status of women with a positive result of a screening test for cervical cancer, applying the Fleiss formula with continuity correction and study power for unmatched cohort studies and cross-sectional studies [22]. With two-tailed testing and type 1 error probability α of 0.05, desired study power of 95% (type 2 error probability β = 0.05), based on the referenced study’s reported prevalence of burden of 1.9% prior to diagnostic procedures, it was determined that a minimum sample of 118 subjects was necessary. To account for possible errors in filling out the questionnaires, it was planned that the minimum sample is to be increased by 10%. Sample size calculation was performed using Epi Info Version 7.2.0.1 software, Centers for Disease Control and Prevention, Atlanta, Georgia.

### 2.5. Data Collection

Self-reported data were collected. Study participants completed an epidemiologic survey and the questionnaires “Process and Outcome Specific Measure, POSM” [23], “Cervical Dysplasia Distress Questionnaire, CDDQ” [24], “Hospital Anxiety and Depression Scale, HADS” [25], and “The Center for Epidemiologic Studies Depression, CES-D” [26] were used.

**Figure 1:**
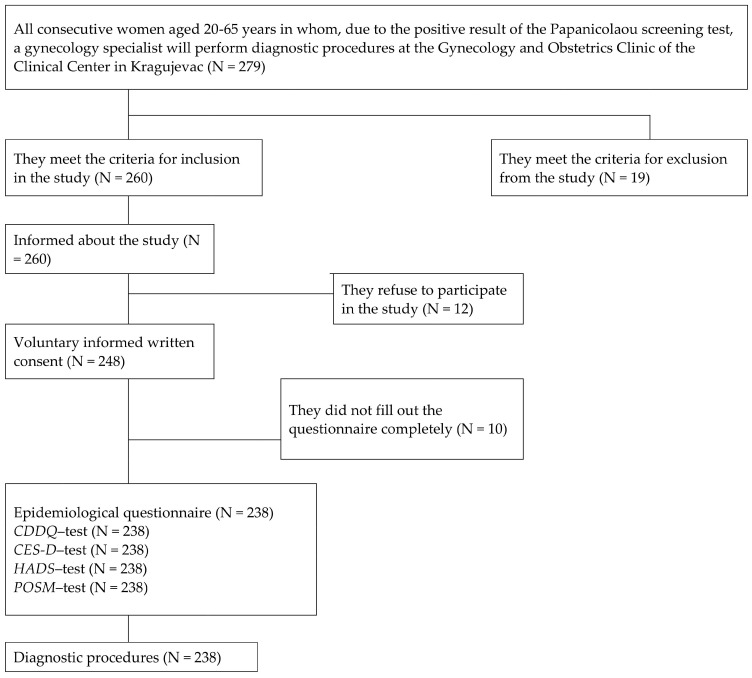
Research flow diagram. Abbreviations: CDDQ (Cervical Dysplasia Distress Questionnaire); CES-D (Center for Epidemiologic Studies Depression scale); HADS (Hospital Anxiety and Depression Scale); POSM (Process and Outcome Specific Measure).

In total, 238 out of 260 women who met the participation criteria were included in this study (Figure 1). The reasons for not accepting or refusing to participate in the study (N = 12) were as follows: lack of time, lack of interest in the study, visual impairment, and insufficient literacy. Some respondents did not fill out the questionnaire during the recruitment for the study, or the questionnaires were not completely filled out (N = 10). For these reasons, these respondents were excluded from the analysis. Response rate was 91.5%.

### 2.6. Instruments

The epidemiologic survey collected information on demographic variables (age, place of residence, educational level, occupation, marital status, body mass index), gynecological and reproductive characteristics (pregnancy, parity, menopause, use of oral contraceptives, abortion history), habits (tobacco smoking, use of alcohol), family history of cervical cancer, etc.

The primary outcome was cervical-cancer-screening-related psychosocial burden. Psychosocial burden was measured using the POSM. The POSM, a specific self-assessment questionnaire for the psychosocial status of women undergoing screening for cervical cancer, was constructed within the TOMBOLA study [23]. Its questions refer to the period between receiving the abnormal screening result and completing the questionnaire. The questions have between five and seven response options on a Likert scale, with a higher score indicating greater psychosocial burden. A recent validation study of the POSM questionnaire [27] reported extraction of 2 factors: factor 1, which contains four items related to worry, and factor 2, which contains three items related to satisfaction with information, support, and changes in the way women feel about themselves. Factor 1 had good reliability (Cronbach’s alpha = 0.769), but the reliability of factor 2 was weaker (0.482). In this research, the two-factor structure of the POSM questionnaire was confirmed, with the internal reliability coefficient of 0.662 for subscale 1 and 0.574 for subscale 2.

The CDDQ is a specific scale for estimating psychological distress in women who received an abnormal Papanicolaou test result in the past year [24]. The scale has four domains: two domains that assess psychological distress related to medical procedures (“Tension and discomfort” and “Embarrassment”) and two domains that assess psychological distress related to the consequences of receiving a positive result of the Papanicolaou test (“Concerns about sexual and reproductive consequences” and “Concerns about health consequences”).

The HADS is a screening tool that is used to estimate the degree of anxiety and depression [25]. For the purposes of this study, respondents’ classification was dichotomized: an HADS subscale’s score ≥ 8 was considered indicative of increased anxiety and/or depression levels, and a score < 8 was considered indicative of low anxiety and/or depression levels.

The CES-D questionnaire is used as a screening test for depression [26], with a score of 16 or higher considered as having a high burden of depressive symptoms.

In this study, the Serbian versions of all used scales (the POSM, CDDQ, HADS, and CES-D questionnaires) were valid and reliable instruments for the assessment of psychological effects in women who are to undergo additional diagnostic procedures due to receiving a positive screening result for cervical cancer [28,29,30,31]. Similar to the original study [27], the structure and reliability of the Serbian version of the POSM were confirmed: factor analysis demonstrated two factors (the worry factor, and the satisfaction with information/support factor), while the Cronbach’s alfa coefficients were 0.662 and 0.574, respectively [28]. The factor analysis of the Serbian version of the CDDQ scale indicated four main components that showed good internal consistency (tension and discomfort = 0.844; embarrassment = 0.864; sexual and reproductive consequences = 0.867; and health consequences = 0.913), while the test–retest reliability coefficients were significant at the 0.01 level for all subscales [29]. The Serbian version of the HADS demonstrated high internal consistency for both subscales (Cronbach’s alpha coefficient for subscale anxiety was 0.862, and for depression, 0.851), and the intra-class correlation coefficients for the two components were significant (0.860 and 0.843, *p* < 0.001) [30]. Overall, the Serbian version of the CES-D scale showed a Cronbach’s coefficient of 0.865, while the Cronbach’s coefficients for the subscales depressed affect, somatic complaints, positive affect, and interpersonal relationship were 0.885, 0.802, 0.851, and 0.593, respectively [31].

### 2.7. Statistical Analyses

Descriptive and analytical statistical methods were used. In order to evaluate characteristics of participants that could be the associated factors of psychosocial burden, i.e., worry and satisfaction with information/support, univariate and multivariate linear regression were performed. The independent variables were all sociodemographic and epidemiological characteristics, habits, personal health history, and family history of cervical cancer. In order to correct for multiple comparisons, Bonferroni correction was used to adjust the significance level. Only variables (potential associated factors) significant at the *p* < 0.05 level based on the univariate linear regression were included in the multivariate linear model. Multivariate linear regression was used to determine the standardized beta coefficient (β), alongside the 95% confidence interval (95% CI) in order to estimate the association of psychological burden and participants’ characteristics. Statistical significance was considered at the *p* < 0.05 level. The SPSS Software (version 20.0, Chicago, IL, USA) was used to perform all statistical analyses.

### 2.8. Ethical Considerations

This study was approved by the Ethics Committee of the Faculty of Medical Sciences, University of Kragujevac (Ref. No.: 01-2176) and by the Ethics Committee of the Clinical Center Kragujevac (Ref. No.: 01-2869). All procedures involving human participants were performed in accordance with the ethical standards of the institutional and/or national research committee and with the 1964 Helsinki declaration and its later amendments or comparable ethical standards. Informed, written, voluntary consent was obtained from all individual participants included in this study before participation in this study, and confidentiality was protected.

## 3. Results

The average age of participants was 46.2 years (±10.5 years); the youngest participant was 23 years old, and the oldest was 65 (Table 1). The total number of years of schooling averaged 11.7 ± 2.6, with a rank of 4–20. The average age at the time of the menarche was about 13 years (±1.7 years); the participant who had her first period the earliest had it when she was 9 years old, and the participant who had her first period the latest had it at 18 years old. The average age at the time of the last menstruation was about 50 years. The woman who had her last menstruation the earliest had it when she was 38 years old, and the woman who had her last menstruation the latest had it when she was 58 years old. The average duration of menopause was about 7 years, with the shortest duration of menopause being 1 year and the longest being 25 years. The participant who was pregnant the earliest was pregnant at 16 years old, and the participant who was pregnant the latest was pregnant when she was 41 years old. The average body mass index was 24.3 (with a range of 15.4–38.9). Psychosocial burden in women undergoing cervical cancer screening according to the POSM scale for the “Worry” domain was an average of 53.56, while for the “Satisfaction with information/support” domain, it was an average of 45.62.

For psychosocial burden (worry, according to the POSM scale) in women with a positive Pap screening test before diagnostic procedures, univariate logistic regression indicated the potential prognostic significance of occupation, use of oral contraceptives, alcohol consumption, discharge as a consequence of the Pap test, psychological distress according to the CDDQ scale, anxiety according to the HADS scale, high burden of depressive symptoms according to the CES-D scale, and insufficient knowledge of the meaning of the term dysplasia/precancerous (Table 2).

For the psychosocial burden (Satisfaction with information/support, according to the POSM scale) in women with a positive Papanicolaou screening test before diagnostic procedures, univariate logistic regression indicated the potential prognostic significance of abortion history, psychological distress according to the CDDQ scale, anxiety according to the HADS scale, and high burden of depressive symptoms according to the CES-D scale (Table 3).

Significant independent predictors of psychosocial burden (Worry, according to the POSM scale) in women with a positive Pap screening test before diagnostic procedures were the use of oral contraceptives (β = −0.174, *p* < 0.001), alcohol use (β = 0.188, *p* < 0.001), anxiety (β = −0.189, *p* = 0.001), high burden of depressive symptoms (β = 0.191, *p* = 0.001), and less knowledge of the meaning of the term dysplasia/precancerous (β = −0.187, *p* < 0.001), according to the multivariate linear regression (Table 4). Significant independent predictor of psychosocial burden (Satisfaction with information/support, according to the POSM scale) in women with a positive Pap screening test before diagnostic procedures was psychological distress according to the CDDQ scale (β = −0.210, *p* = 0.001).

## 4. Discussion

This study reports on independent predictors of higher psychosocial burden before diagnostic procedures (colposcopy/biopsy/endocervical curettage) in women who received an abnormal Pap screening test result, i.e., for worry, those include the use of oral contraceptives, alcohol consumption, anxiety, depression, and insufficient knowledge about the meaning of the term dysplasia/precancerous, while for high satisfaction, with information/support, the predictor was lower psychological distress related to medical procedures/concern about sexual and reproductive consequences/concern about health consequences.

Although the use of oral contraceptives is linked with the risk of cervical cancer, there are no data in the available literature regarding a connection with cervical cancer screening. A low screening rate was described among women in Ouagadougou (Burkina Faso), whereby some risk behavior factors (such as oral contraceptive use, etc.) and limited knowledge about cervical cancer were associated with cervical cancer screening practice [32]. The inverse association of oral contraceptive use with worry in women with a positive Pap screening test before diagnostic procedures in our study could be attributed to frequent visits to the gynecologist and, consequently, a better level of knowledge of screening procedures and better information/support from health professionals.

Apart from high alcohol consumption possibly increasing the risk of cervical cancer high risk-HPV persistence in women, several studies found that alcohol consumption was associated with lower rates of cervical cancer screening participation [33]. Although there are no similar results in the available literature, this study showed an association between alcohol use and worries in women participating in cervical cancer screening, which could be related to the high frequency of alcohol consumption in the female population in Serbia, where alcohol use is considered as a socially acceptable behavior and a part of tradition, customs, and culture [34].

In women with low-grade abnormal cervical cytology involved in the TOMBOLA trial in the United Kingdom, all worries (about cervical cancer, sex, future fertility, and general health) during the follow-up were related to the HADS-measured anxiety and, to a lesser extent (worries about cervical cancer, sex, and general health), with the HADS measured depression, while the correlations between satisfaction with information/support and the HADS scores for anxiety and depression were lower [27]. In our study, anxiety was associated with worry in women with a positive Pap screening test before diagnostic procedures, which could be explained by the uncertainty of the screening procedures, fear of diagnosis of malignancy together with complicated surgeries, by pain and discomfort during the examination (namely, discharge as a consequence of the Papanicolaou test’s screening procedure was significantly associated with worry, although only according to the univariate analysis), relatively little experience with the organized cervical cancer screening program in the population of Serbia, etc. In women attending the hospital-based colposcopy clinic for abnormal cervical cytology in the TOMBOLA study [35], the likelihood of worry about cervical cancer was associated with a history of depression (self-reported depression) according to the univariate analysis, but not with worry about future fertility and worry about having sex. This finding was confirmed in our study, whereby the high burden of depressive symptoms was associated with worry, while some other authors did not report on significance of depression after a notification of an abnormal Pap smear result [36].

In this study, better knowledge of the meaning of the term dysplasia/precancerous was associated with a lower level of cervical cancer screening-related worry. One systematic review that investigated pathways between health literacy and cervical cancer screening, found inconsistent findings in terms of the relationships between health literacy, psychosocial factors, and cervical cancer screening, although limited research suggested a potential link between health literacy and cervical cancer screening through cervical cancer knowledge [37]. A qualitative study among women attending a colposcopy clinic for the first time in Cape Town, South Africa found that low peer encouragement was a factor identified as a potential barrier to access, while the impact of insufficient information from health professionals about colposcopy referral and anxiety during waiting times between obtaining results and scheduling a colposcopy was suggested [38]. In addition to consideration that the colposcopy-related worries have been thought to be due to the lack of knowledge about cervical cancer screening and colposcopy [39], some authors reported that the low knowledge scores before colposcopy were positively associated with educational level [40]. After receiving abnormal cervical smear results, women of lower socioeconomic status in Canada did not fully understand the information about their result [41]. According to the univariate analysis in this study, occupation (professional) was negatively associated with worry, although this association was not independent. The question remains as to how many women with a lower education level undergo additional diagnostic procedures, since the level of education in this study was not significantly related to worry or satisfaction with information/support.

In this study, a lower satisfaction score with information/support was significantly associated with a higher level of psychological distress by the CDDQ scale (that is, with tension and discomfort regarding the screening test, as well as with concerns regarding sexual and reproductive consequences, and health consequences). The finding that being dissatisfied with received support is a predictor of distress is compatible with previous studies which indicated that dissatisfaction with social support (from hospital staff, family, or friends) was associated with distress and anxiety [12,42]. According to the univariate analysis, abortion history was negatively associated with satisfaction with information/support, which could also be related either to insufficient information/support provided by health professionals or to a still significant representation of abortion as a method of contraception in Serbia [43].

This study highlighted that women experience psychological burden in relation to further diagnostic evaluation of pre-cancerous cell changes in the cervix and provided better knowledge about the variables that contribute to the elevated psychosocial burden prior to colposcopy/biopsy. And it could help direct interventions for reducing psychosocial burden prior to diagnostic procedures.

### 4.1. Implications

Assessing the levels and identifying associated factors of the psychosocial burden among women before undergoing additional diagnostics after receiving an abnormal cervical cancer screening result is of great public health importance. Firstly, a considerable number of women could be experiencing psychosocial burden, especially in countries where the implementation of organized screening for cervical cancer is still underway. Namely, since there has been no established organized cervical cancer screening in previous years, many women did not participate in the screening and are just beginning to become familiar with the corresponding procedures. Furthermore, this study found that women who are at the highest risk of psychosocial burden have low knowledge of the meaning of the term dysplasia/precancerous, have psychological distress after screening tests, have anxiety, depression, use alcohol, and lack oral contraceptive use. These women may represent particularly vulnerable subgroups of the screening population, especially when they are to undergo additional diagnostic examinations. Strategies are needed to minimize the psychosocial burden among women before undergoing additional diagnostics due to an abnormal screening smear result. Interventions that focus particularly on women’s understanding of the screening smear results and the meaning of the term dysplasia/precancerous seem to offer the best opportunity to reduce the psychosocial burden.

This study revealed that women who seem satisfied with the information/support they received regarding cervical cancer screening have a lower psychosocial burden. This is indicated by the finding of this study that women who were satisfied with the information/support they received regarding cervical cancer screening were women who used oral contraceptives, and it suggests that any visits to the doctor give a chance to obtain the necessary information and in relation to other issues, including cervical cancer screening. The finding that women were often worried (about cervical cancer and any additional treatment required, general health, future fertility, having sex) suggests that it would be appropriate to provide to women the information that directly addresses their concerns related to cervical cancer screening prior to receiving the smear result. A recent study that examined the psychological distress experienced by patients with an initial diagnosis of abnormal Pap smears in Germany showed that more than 68% of women primarily obtain information from their gynecologist, which underscores the importance of the doctor–patient relationship [44]. Apart from that, another way could be the example of the Tailored Communication for Cervical Cancer Risk intervention [45], an efficacious telephone-based intervention that targets five psychosocial barriers to women scheduled for a follow-up appointment after an abnormal Papanicolaou test result, including lower knowledge and risk perceptions, negative beliefs and expectancies, interfering affect, values and goals, and self-regulatory skills. How this information should be provided would be an issue for further research, but it would best be provided by the healthcare professionals at the time of the smear appointment. This could potentially mitigate the psychosocial burden associated with all cervical-cancer-screening procedures.

### 4.2. The Strengths and Limitations of the Study

No studies appear to have investigated the psychosocial impact of undergoing additional diagnostic procedures (colposcopy/biopsy/endocervical curettage) in women who received an abnormal result of the Papanicolaou screening test. In addition, factors associated with cervical cancer screening and diagnostics—specifically worry and satisfaction with information/support—were identified for the first time. The strength of this study is the use of a cervical-cancer-screening-specific measure of psychosocial burden in women (POSM). Additionally, the POSM and all other questionnaires were linguistically adapted and validated for the population in this study. Also, because women were recruited from the only clinic in this county, it could be assumed that the women who consented to the study are typical representatives of women attending cervical cancer screening in the entire area.

The limitations of this study include its cross-sectional design (with well-known limitations regarding the ‘ecological fallacy’), use of self-report questionnaires, possibility of information bias (although the survey was anonymous) and response bias (because women suffering from psychosocial burden may not have responded to additional diagnostic procedures or may have chosen not to participate in the research, or may have been more or less likely to answer the survey), and lack of information on some other characteristics of women (such as socio-economic status, etc.)

## 5. Conclusions

A number of women participating in cervical cancer screening require follow-up due to an abnormal Pap smear. However, little is known about the psychosocial burden of women undergoing further diagnostic procedures. This study showed that the psychosocial burden before diagnostic procedures is more pronounced in women with low knowledge of the meaning of the term dysplasia/precancerous, who consume alcohol, who have a higher level of psychological distress after the screening test, and those with a higher score for anxiety and depression. These women may represent a particularly vulnerable group among those referred for further diagnostic procedures after having received an abnormal result of the Papanicolaou screening test. Providing uniform and explicit information about cervical cancer screening to better understand the meaning of the term dysplasia/precancerous and identifying which women are at risk of psychosocial burden may help protect against this potential harm in the population of women who received a positive cervical-cancer-screening result and may facilitate their intention to undergo further diagnostic procedures.

## Figures and Tables

**Table 1:** Psychosocial burden of study participants (by POSM scale): descriptive statistics.

Variables	Mean ± SD, Min–Max
Age (years)	46.2 ± 10.5, 23–65
Total number of years of schooling	11.7 ± 2.6, 4–20
Menarche age	13.3 ± 1.7, 9–18
Age of menopause	49.4 ± 4.2, 38–58
Duration of menopause	6.7 ± 5.6, 1–25
Age at first pregnancy	23.7 ± 4.3, 16–41
Body mass index	24.3 ± 4.2, 15.4–38.9
POSM subscales	Mean ± SD (Range), Min–Max
- Worry	43.56 ± 11.94 (1–100), 15.87–72.22
- Satisfaction with information/support	45.62 ± 14.04 (1–100), 16.67–87.50

POSM (Process and Outcome Specific Measure); SD (Standard Deviation).

**Table 2:** Psychosocial burden (Worry, by POSM scale) by characteristics of study participants.

Variables	β (95%CI)	*p*
Age	0.032 (−0.767; 1.415)	0.559
Place of residence (Urban)	−0.025 (−3.521; 2.197)	0.649
Educational level (Faculty)	−0.025 (−3.793; 2.366)	0.649
Occupation (Professional)	−0.128 (−2.116; −0.201)	0.018 *
Marital status (With partner)	0.083 (−0.694; 5.713)	0.124
Pregnancy (Ever)	−0.033 (−4.970; 2.605)	0.540
Children (Yes)	−0.007 (−3.724; 3.235)	0.890
Menopause (Yes)	−0.006 (−2.733; 2.445)	0.913
Oral contraceptive use (Ever)	−0.132 (−7.774; 0.876)	0.014 *
Abortion history (Yes)	0.029 (−2.222; 3.711)	0.622
Tobacco use (Ever)	0.016 (−2.183; 2.930)	0.774
Alcohol use (Ever)	0.112 (0.118; 6.817)	0.038 *
Body Mass Index (≥25 kg/m^2^)	0.069 (−0.894; 4.271)	0.199
Family history of cervical cancer (Yes)	0.015 (−3.625; 4.856)	0.775
Consequences of the screening procedure (Papanicolaou test)		
Pain	0.022 (−3.439; 5.274)	0.679
Bleeding	0.052 (−1.767; 5.183)	0.334
Discharge	0.011 (0.150; 6.468)	0.040 *
Psychological distress by CDDQ scale	0.243 (3.189; 7.902)	<0.001 *^,^**
CDDQ subscales		
Tension and discomfort	0.157 (1.005; 5.102)	0.004 *
Embarrassment	0.151 (0.591; 3.304)	0.005 *
Sexual and reproductive consequences	0.198 (1.813; 5.854)	<0.001 *^,^**
Health consequences	0.171 (0.903; 3.761)	0.001 *^,^**
HADS—Anxiety score (8–21 points)	0.298 (0.552; 1.123)	<0.001 *^,^**
HADS—Depression score (8–21 points)	0.285 (0.552; 1.102)	<0.001 *^,^**
CES-D-High burden of depressive symptoms (≥16 points)	0.284 (0.232; 0.491)	<0.001 *^,^**
CESD subscales		
Somatic complaints	0.347 (0.029; 0.068)	<0.001 *^,^**
Positive affect	0.076 (−0.109; 0.667)	0.159
Depressed affect	0.226 (0.332; 0.891)	<0.001 *^,^**
Interpersonal relationship	0.146 (0.410; 2.528)	0.007 *
Knowledge of the meaning of the term dysplasia/precancerous	−0.205 (−7.524; −2.456)	<0.001 *^,^**
Mode of obtaining notification of abnormal Papanicolaou result	−0.014 (−1.064; 0.815)	0.795

POSM (Process and Outcome Specific Measure); CDDQ (Cervical Dysplasia Distress Questionnaire); HADS (Hospital Anxiety and Depression Scale); CES-D (Center for Epidemiologic Studies Depression scale). β (standardized regression coefficient beta); 95%CI (Confidence Interval); *p* (value by univariate linear regression); * Nominally significant (*p* < 0.05); ** Characteristic that remains significant after Bonferroni correction for multiple comparisons.

**Table 3:** Psychosocial burden (Satisfaction with information/support by POSM scale) by characteristics of study participants.

Variables	β (95%CI)	*p*
Age	0.032 (−0.894; 1.673)	0.551
Place of residence (Urban)	0.049 (−1.803; 4.918)	0.363
Educational level (Faculty)	−0.054 (−5.476; 1.763)	0.314
Occupation (Professional)	0.044 (−0.667; 1.603)	0.418
Marital status (With partner)	0.028 (−2.801; 4.761)	0.610
Pregnancy (Ever)	−0.051 (−6.592; 2.315)	0.346
Children (Yes)	−0.027 (−5.115; 3.070)	0.623
Menopause (Yes)	−0.005 (−3.192; 2.900)	0.925
Oral contraceptive use (Ever)	0.026 (−3.103; 5.082)	0.635
Abortion history (Yes)	−0.119 (−7.381; −0.181)	0.040 *
Tobacco use (Ever)	−0.035 (−3.892; 2.030)	0.524
Alcohol use (Ever)	−0.029 (−5.005; 2.841)	0.588
Body Mass Index (≥25 kg/m^2^)	0.052 (−1.567; 4.517)	0.341
Family history of cervical cancer (Yes)	0.092 (−0.649; 9.290)	0.088
Consequences of the screening procedure (Papanicolaou test)		
Pain	0.023 (−4.024; 6.277)	0.673
Bleeding	0.073 (−1.262; 6.904)	0.175
Discharge	−0.053 (−5.601; 1.868)	0.326
Psychological distress by CDDQ scale	−0.273 (−10.077; −4.578)	<0.001 *^,^**
CDDQ subscales		
Tension and discomfort	−0.172 (−6.361; −1.554)	0.001 *^,^**
Embarrassment	0.003 (−1.574; 1.654)	0.961
Sexual and reproductive consequences	−0.220 (−7.373; −2.641)	<0.001 *^,^**
Health consequences	−0.379 (−7.664; −4.505)	<0.001 *^,^**
HADS—Anxiety score (8–21 points)	−0.199 (−1.003; −0.314)	<0.001 *^,^**
HADS—Depression score (8–21 points)	−0.136 (−0.808; −0.102)	0.012 *
CES-D-High burden of depressive symptoms (≥16 points)	−0.137 (−0.363; −0.048)	0.011 *
CESD subscales		
Somatic complaints	−0.088 (−0.706; 0.066)	0.101
Positive affect	−0.102 (−0.896; 0.016)	0.058
Depressed affect	−0.127 (−0.738; −0.068)	0.018 *
Interpersonal relationship	−0.003 (−1.301; 1.218)	0.949
Knowledge of the meaning of the term dysplasia/precancerous	0.087 (−0.546; 5.523)	0.108
Mode of obtaining notification of abnormal Papanicolaou result	0.051 (−0.573; 1.636)	0.344

POSM (Process and Outcome Specific Measure); CDDQ (Cervical Dysplasia Distress Questionnaire); HADS (Hospital Anxiety and Depression Scale); CES-D (Center for Epidemiologic Studies Depression scale). β (standardized regression coefficient beta); 95%CI (Confidence Interval); *p* (value by univariate linear regression); * Nominally significant (*p* < 0.05); ** Characteristic that remains significant after Bonferroni correction for multiple comparisons.

**Table 4:** Associated factors of psychosocial burden (by POSM subscales) among women with abnormal Papanicolaou smear results before diagnostic procedures.

	Worry		Satisfaction with Information/Support	
Variables ^†^	β (95%CI)	*p*	β (95%CI)	*p*
Oral contraceptive use (Ever)	−0.174 (−8.857; −2.514)	<0.001 *^,^**		
Alcohol use (Ever)	0.188 (2.863; 8.949)	<0.001 *^,^**		
Psychological distress by CDDQ scale			−0.210 (−8.859; −2.494)	0.001 *^,^**
HADS—Anxiety score (8–21 points)	0.189 (0.227; 0.838)	0.001 *^,^**		
CES-D—High burden of depressive symptoms (≥16 points)	0.191 (0.103; 0.383)	0.001 *^,^**		
Knowledge of the meaning of the term dysplasia/precancerous	−0.187 (−6.916; −2.198)	<0.001 *^,^**		

POSM (Process and Outcome Specific Measure); CDDQ (Cervical Dysplasia Distress Questionnaire); HADS (Hospital Anxiety and Depression Scale); CES-D (Center for Epidemiologic Studies Depression scale). β (standardized regression coefficient beta); 95%CI (Confidence Interval); *p* (value by multivariate linear regression). ^†^ (only statistically significant independent associated factors are presented). * Nominally significant (*p* < 0.05); ** Characteristic that remains significant after Bonferroni correction for multiple comparisons.

## Data Availability

Data are contained within the article.
